# Binge Drinking in Young University Students Is Associated with Alterations in Executive Functions Related to Their Starting Age

**DOI:** 10.1371/journal.pone.0166834

**Published:** 2016-11-18

**Authors:** Diana Salas-Gomez, Mario Fernandez-Gorgojo, Ana Pozueta, Isabel Diaz-Ceballos, Maider Lamarain, Carmen Perez, Pascual Sanchez-Juan

**Affiliations:** 1 Gimbernat-Cantabria Research Unit (SUIGC), University Schools Gimbernat-Cantabria, Attached to the University of Cantabria, Torrelavega, Spain; 2 University Schools Gimbernat-Cantabria, Attached to the University of Cantabria, Torrelavega, Spain; 3 Service of Neurology, University Hospital “Marqués de Valdecilla”, University of Cantabria (UC), CIBERNED, IDIVAL, Santander, Spain; Technion Israel Institute of Technology, ISRAEL

## Abstract

Our aim was to evaluate whether or not alcohol consumption in the form of binge drinking is associated with alterations of memory and executive functions in a population of university students. At the same time, we have studied the role of potential modulating factors, such as the APOE genotype or physical exercise.University students enrolled in academic year 2013–2014 at Escuelas Universitarias Gimbernat-Cantabria, affiliated with the University of Cantabria, were invited to participate in the study. We gathered sociodemographic data and details regarding the lifestyle of 206 students (mean age 19.55 ± 2.39; 67.5% women). We evaluated memory and executive functions via a series of validated cognitive tests. Participants were classified as binge drinkers (BD) and non-BD. Using Student's t-distribution we studied the association between cognitive tests and BD patterns. Multivariate analyses were carried out via multiple linear regression. 47.6% of the students were found to be BD. The BD differed significantly from the non-BD in their results in the executive functions test TMT B (43.41 ± 13.30 vs 37.40 ± 9.77; p = 0.0003). Adjusting by age, sex, academic records, age at which they started consuming alcohol, cannabis consumption, level of physical activity and other possible modifying variables, the association was statistically significant (p = 0.009). We noticed a statistically significant inverse correlation (Pearson’s r^2^ = -0.192; p = 0.007) between TMT B and starting age of alcohol consumption. Differences were observed in another executive functions test, TMT A, but only in the group of women (19.73±6.1 BD vs 17.78±5.4 non-BD p = 0.05). In spite of the young age of our participants, BD was associated with a lower performance in the executive functions test (TMT B). These deficits were related to the age at which they started drinking alcohol, suggesting an accumulative effect.

## Background

Among young people in the west there is an alcohol consumption pattern that is characterised by large amounts being drunk in short periods of time following periods of abstinence. This is known as "binge drinking" (BD). [1] Globally, BD is highly prevalent in the younger population, involving up to 40% of university students, depending on the country. [2–4] A recent study in Spain found that 37% of first-year university students consumed large amounts of alcohol, and 12.2% were classified as BD. [5] Alcohol consumption in countries like Spain starts very early, with more than fifty percent of teenagers between the ages of 14 and 18 acknowledging at least one BD session in the last month and a starting age for alcohol consumption of 13.9 years of age. [6]

On the whole, in our society there is awareness of the consequences of chronic alcohol consumption, but there is not such a clear perception of the risks posed by occasional consumption. However, studies carried out on animals indicate that BD implies greater damage to the central nervous system than regular consumption. Likewise, greater neuropsychological repercussions have been described in BD, as compared to regular drinkers. [7]

Although alcohol consumption is legally prohibited for adolescents, there is a high level of permissiveness in our social sphere, as shown by the early starting age. [6] Due to its immaturity, during adolescence the brain is particularly sensitive to the effects of alcohol. Given the high neuroplasticity of hippocampus, and that the prefrontal cortex is the area of the brain that take the longest to develop, it is to be expected that these structures are especially vulnerable in the younger population. [8,9] This is coherent with the fact that the cognitive and affective pattern found in youths who binge drink is mainly related with alterations of memory and executive functions. Despite the extremely high prevalence and evidence of damage caused by binge drinking on young people's brains, its effects on cognitive functions at early ages have not been studied systematically in larger populations until quite recently and its consequences in the long term are broadly unknown. S1 Table includes a literature review of studies assessing the effect of binge drinking on cognitive performance using neuropsychological tests in young population. Even though many of these studies are underpowered, with only one reaching a population sample over 100 individuals (Parada 2012), it has been observed that young people who binge drink carry out neuropsychological tasks involving attention span and planning less effectively [10,11] and are associated with a worse verbal declarative memory [12–14] and working memory. [15] Likewise, several neuroimaging studies have found structural and volumetric differences in the prefrontal cortex in young BDs compared to non-BDs. [16–18] Moreover, there are genetic factors, such as the E4 allele of the *APOE* gene, which has been demonstrated to potentially interact with alcohol consumption in individuals with cognitive impairment. [19–22] However, this relationship has not been studied in young individuals who binge drink. Nor have other potential modulators of the effect of BD been studied, such as physical exercise, which has positively correlated with cognitive performance. [23]In our project we plan to evaluate whether BD is associated with alterations of the memory and executive functions in a group of university students. Likewise, we intend to study the role of potential modulating factors, such as the APOE genotype or physical exercise.

## Patients and Methods

The study included all university students enrolled in academic year 2013–2014 at Escuelas Universitarias Gimbernat-Cantabria, affiliated with the University of Cantabria. Exclusion criteria were: a history of severe craneoencephalic trauma, neurological diseases, dyslexia, colour blindness, difficulties with the Spanish language and subjects with sensory deficits.

The study was reviewed and approved by our institutional review board (Comité Ético de Investigación de Cantabria) before the study began. All participants signed an informed consent before entering the study. At study baseline all participants were over 18 years of age with the exception of 8 individuals aged 17. They were all first year students that turned 18 during the academic course, there were no additional participants under the age of 18 whose birthday did not fall within the study period that lasted two years. In agreement with our review board, the 8 minor participants signed the written informed consent document during the study period once they turned 18 years. Once the informed consent was signed, an interview took place divided into two individual sessions lasting 30 minutes. Firstly, participants filled out a questionnaire regarding the consumption of alcohol and other drugs like cannabis, sociodemographic information and lifestyle habits via the international physical activity questionnaire (IPAQ). [24] The alcohol consumption questionnaire was based on the BD criteria established by the National Institute for Alcohol Abuse and Alcoholism, and included specific questions about average alcohol unit intake and speed of consumption, allowing us to define the BD pattern. [25]. They were then given a battery of tests, validated for the study of a young population and aimed at assessing memory and executive functions. The assessors were given prior training in order to administer and evaluate correctly the cognitive tests in a standardised way. A logical memory test was given (WMS-III) [26,27] together with the CERAD word list [28–30] to study episodic verbal memory; the Rey-Osterrieth complex figure test (copy and recall) [31] to test for constructional apraxia and differed visual memory; the digit span test WAIS-III [26,27] to test working memory, attention span and concentration; the colour word test (STROOP) [32] to check capacity to inhibit automatic response and the Trail Making Tests (TMT) A and B [33] to evaluate visual-motor speed (part A) and attention and mental flexibility (part B). Blood samples were taken from all participants to extract DNA and for subsequent genotyping of the polymorphism of the APOE*E4 via Taqman assays.

### Statistical analysis

A univariate analysis was carried out using Student's t-distribution to evaluate the effect of our main variable (BD or non-BD) on the scores of the cognitive tests. To do this, BD were considered to be those who consumed 5 or more alcoholic drinks in the space of two hours (4 for females) in an average month, following the BD criteria defined by the National Institute on Alcohol Abuse and Alcoholism. [25] Likewise, the two main covariates were categorised: a) *APOE* genotype, depending on whether or not they had the E4 allele; and b) physical activity (at least moderate or sedentary) following the criteria described by the IPAQ. [24] Multivariate analyses were carried out using multiple linear regression including, together with BD pattern, possible modifying variables or confounders of the effect.

The statistical analysis of the data was carried out using SPSS 19.0 (Statistical Product and Service Solutions IBM SPSS Statistics 19.0 2010).

## Results

The final sample included 206 individuals, with a mean age of 19.55 ± 2.39 years, of which 67.5% were women. Only 2 foreign students were excluded due to their lack of understanding of the Spanish language. 96.6% of the students said that they had consumed alcohol at some time, with an average starting age of 15.19 ± 1.36 years. 53.6% stated that they had got drunk at least once in the last month and 57.3% declared that they had had problems with memory loss following excessive alcohol consumption. 47.6% of the students were found to be BD. Table 1 shows the most relevant sociodemographic characteristics and summarises the lifestyle habits questionnaire as far as alcohol and cannabis consumption are concerned.

**Table 1:** Sociodemographic characteristics.

	Total (N = 206)*	BD (N = 98)	Non-BD (N = 106)	P-value
**Mean age ± SD (years)**	19.5±2.4	19.4±2.3	19.7±2.4	0.35
**Percentage of women**	67.5	64.3	69.8	0.46
**Academic record mean ±SD**	6.3±1.4	6.4±1.2	6.3±1.4	0.87
**Mean days per month that you consume 5 or more alcoholic drinks ±SD**	2.0±2.1	3.54±1.9	0.6±1.2	<0.0001
**Units of alcohol that you usually drink when you go out (%)**	3–4 (42.4)5-6 (13.6)7-9 (17.5)	3–4 (23.5)5-6 (22.4)7-9 (31.6)	3–4 (14.2)5-6 (5.7)7-9 (4.7)	<0.0001
**Mean age at onset of alcohol consumption ±SD (years)**	15.2±1.4	14.7±1.2	15.7±1.3	<0.0001
**An episode of drunkenness in the last month (%)**	53.6	100.0	15.1	<0.0001
**“Black outs” after alcohol consumption (%)**	57.3	78.6	37.7	<0.0001
**Percentage of students that consume cannabis (>5 days per month)**	25.7	40.70	10.60	<0.001
**Mean age of onset of cannabis use ±SD (years)**	16.4±2.6	16.8±1.7	15.4±4.2	0.07
**Insufficiently active vs minimally active**	70.0 vs 30.0	73.5 vs 26.5	67.9 vs 32.1	0.38

* There were two individuals with missing information

Table 2 shows the results of the neuropsychological tests in both BD and non-BD students. We found statistically significant differences in the results of the TMT B test. Whereas the students who binge drink took an average of 43.41 ± 13.30 seconds to complete the test, students who do not binge drink took 37.40 ± 9.77 seconds (p = 0.0003) (Bonferroni corrected p = 0.036). After adjustment according to age, sex, academic records, alcohol consumption starting age, cannabis consumption, level of physical activity and other possible modifying variables or confounders, such as the time of day, the researcher and the day of the week upon which tests were carried out, the association between TMT B and students who binge drink maintained its statistical significance (p = 0.009). The result of the TMT A tests was 18.88 ± 5.76 seconds in the youths who binge drink and 17.67 ± 5.51 seconds for those who do not, not showing statistically significant differences (p = 0.13). However, after carrying out the multivariate analysis, adjusting by the same variables as in the previous analysis, a significant association was found between the BD consumption pattern and TMT A (p = 0.05). Significant differences were not found for any other neuropsychological tests in relation with BD.

**Table 2:** Results of the neuropsychological tests in both binge drinking and non-binge drinking students.

	BD(N = 98) mean ±SD	Non-BD(N = 106) mean ±SD	P-value	P -value*
**Logical memory WAIS-III (LMW) immediate recall**	23.96±6.18	23.55±6.23	0.64	0.58
**LMW delayed recall**	26.14±6.24	26.57±6.71	0.64	0.25
**CERAD immediate recall**	25.70±2.47	25.40±3.22	0.45	0.73
**CERAD deferred words**	9.10±1.05	9.02±1.40	0.66	0.21
**CERAD recognition list**	19.94±0.28	19.74±1.34	0.14	0.36
**Rey figure copy**	35.81±0.88	34.45±1.94	0.09	0.27
**Rey figure delayed visual memory**	23.96±5.65	25.20±5.52	0.11	0.28
**Digits forward (raw score)**	9.81±2.38	9.69±2.02	0.70	0.67
**Digits backward (raw score)**	6.81±2.10	7.69±9.56	0.24	0.21
**Stroop test interference**	51.54±8.49	52.35±8.91	0.51	0.28
**Trail making test A**	18.88±5.76	17.67±5.51	0.13	0.052
**Trail making test B**	43.41±13.30	37.40±9.77	0.0003	0.009

BD binge drinkers

* P-values adjusted by: age, sex, academic record, day of week, time at which the test was performed, person carrying out the examination, physical activity level, cannabis use, and age at which they started drinking.

13.9% of the population studied were carriers of the APOE*E4. We did not find significant differences in terms of memory or executive function performance of carriers and non-carriers; although differences in Rey Figure copy reached nominal significance between the two groups (p = 0.01), this was not mantained after Bonferroni correction (p = 0.12) (S2 Table). 67.5% of the population was female; differences were not found in the neuropsychological tests associated to gender. (S3 Table). Nor did the level of physical activity correlated with the test results in our sample (S4 Table). In a post hoc analysis we explored the effect of binge drinking on TMT A and TMT B according to gender. In Table 3 we can see that whereas in TMT B the extent of the effect is similar for both sexes, in TMT A we can observe that the differences only affect the group of females. Finally, we studied the link between alcohol consumption starting age and performance in these tests.

**Table 3:** Effect of Binge Drinking on TMT A and B stratified by gender.

		TMT Amean ±SD	p-value	TMT Bmean ±SD	p-value
**Men**	**BD (N = 35)**	17.37±4.89	0.78	43.40±15.77	**0.019**
	**Non- BD (N = 32)**	17.41±5.73		36.03±8.34	
**Women**	**BD (N = 63)**	19.73±6.1	**0.05**	43.41±11.9	**0.005**
	**Non-BD (N = 73)**	17.78±5.4		38.00±10.33	

BD Binge Drinkers; TMT A Trail making test A; TMT B Trail Making Test B

**Fig 1:**
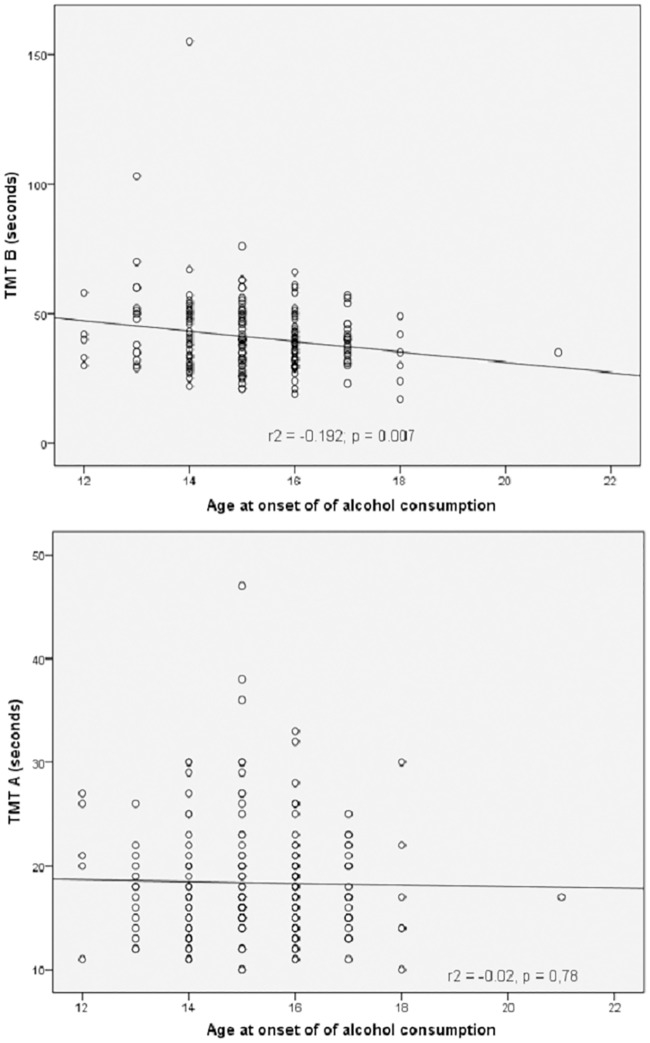
Correlation between TMT A and TMT B with age at onset of alcohol consumption.

Fig 1 shows scatter diagrams demonstrating the results of TMT A and TMT B and alcohol consumption starting age. We noticed a statistically significant inverse correlation (Pearson’s r^2^ = -0.192, p = 0.007; Spearman’s rho = -0.149, p = 0.035) between TMT B and starting age of consumptiom; that is, the earlier the students started to drink alcohol, the longer they took to do the tests, reflecting a lesser degree of cognitive flexibility.

## Discussion

The main aim of this research was to evaluate the cognitive effects of binge drinking on university students, above all on their executive functions and memory. In our analysis we observed that the students who binge drink obtained worse results in the TMT B test, which is a task that measures executive functions focusing on attention and mental flexibility. Students who binge drink performed worse in the TMT B, carrying out the test on average six seconds slower than those who do not binge drink. These results continued to be significant after adjusting them according to the main co-variates and potential confounders. In the multivariate analysis, the statistically significant association of the BD sample with another executive function test such as TMT A, which specifically measures visual motor speed and attention, was also observed. To gain a better understanding of this association we carried out a stratified analysis according to gender which showed that the association with TMT A mainly affected the female subgroup.

The effects of alcohol on executive functions are well documented; however, the majority of studies were carried out on middle-aged subjects. [34–36] The data obtained in our analysis are consistent with other authors who reported a repercussion of alcohol in young people and adolescents on several tests that assess executive functions. [18, 37–42] In our univariate analysis we found that TMT B was the only executive function test associated with the BD pattern of alcohol consumption. We hypothesise that TMT B is the most sensitive test to capture the early effects of binge drinking in young students’ cognition. In a study carried out on 91 alcohol drinkers, with an average age of 19.4, it was observed that heavy alcohol consumption, measured by the concentration on the breath, was associated with a poorer performance in TMT B, and chronic consumption, estimated by the number of years drinking, was associated, regardless of how heavy the consumption was, with a poorer performance in TMT A and TMT B. The authors hypothesised that TMT B, which evaluates more complex and specific tasks relating to executive functions, such as mental agility, would be more sensitive to the effects of alcohol, as opposed to TMT A, which evaluates more basic aspects, such as visual motor coordination, which are not directly dependent on the dorsolateral prefrontal cortex. [43] A similar pattern can be seen in our study, where the association with TMT A only reaches the statistical significance in the female subgroup. These differences in function of each gender may be explained by the theory that the female brain is more vulnerable to alcohol than the male brain. [10,40–45] However, these results are controversial, and other studies have not found any interaction between alcohol consumption and gender and cognitive performance. [14,15,46] It has been argued that not taking into account factors that may lead to confusion, such as when the alcohol consumption started, may be related to these discrepancies. [37] Therefore, one advantage of our study is that a comprehensive multivariate analysis has been carried out, allowing the adjustment of our main results by a multitude of co-variables and potential confounders. Moreover, our analysis, including 206 individuals with neuropsychological assessment, is the largest sample of these characteristics that has been reported to date. S1 Table summarises the publications that have evaluated the cognitive effects of binge drinking in young people until now.

Based on the results obtained by our study and previous studies, we can interpret that binge drinking in young people would have a predominant effect upon executive functions, particularly cognitive flexibility, which is the most specific aspect captured by TMT B, compared to the other executive function test that we used. [16,37] This function would be the most susceptible to being affected by early alcohol consumption through binge drinking. The area of the brain which most specifically correlates with mental flexibility is the dorsolateral prefrontal cortex. Therefore, the changes in this area of the brain reported in previous MRI studies in young people who binge drink are coherent with our results. [16,17,18] The preferential damage to this area could be related to a greater sensitivity to the toxicity caused by alcohol, as mentioned earlier. [8,9,47,48] Moreover, these data concur with the hypothesis that alcohol would affect those areas that mature later on in human development, as the prefrontal cortex is the last part of the brain to fully develop. [3,49,50]

Together with the association between binge drinking and the TMT B results, as well as those of the TMT A to a lesser extent, the other main finding of our research is the strong inverse correlation between alcohol consumption starting age and the time taken to carry out the TMT B. These data suggest that the damage caused by heavy intermittent alcohol consumption could have an accumulative effect at an early age; even during relatively short time periods, as our population had only been consuming alcohol for 4.22 years on average.

A possible modulation factor of the effects of alcohol on cognitive functions is the APOE*E4 allele. Our analysis does not prove the existence of an interaction between the *APOE* genotype and binge drinking where its effect on cognition is concerned. Even if our study is the first to pose this question regarding young people who binge drink, previous publications have already shown the interaction between prolonged alcohol consumption and the APOE genotype in subjects over 60 years of age. [21] Likewise, other authors have observed that patients with Alzheimer's carrying the APOE*E4, who also had a history of heavy alcohol consumption, developed the disease years earlier than APOE*E4 carriers who did not have a history of heavy alcohol consumption. [22] The lack of evidence of interaction in our study could initially be due to a limited statistical power for the analysis, as only 13.9% of the sample carried APOE*E4. On the other hand, the effect on the APOE*E4 cognitive tests is controversial in young people and in certain studies it has been found that the carriers of this allele, which is the main genetic risk factor for cognitive impairment, even perform better than the non-carriers. [51] This phenomenon, known as antagonistic pleiotropy, would complicate the interpretation of a possible interaction with the BD pattern. The other modulation factor assessed in our study was physical exercise. This factor has been consistently associated with neuroprotection. [23] However, in our analysis we found no significant effect on any cognitive domain (S4 Table), nor a modulation of the effect of alcohol on cognition. A limited statistical power to detect the effect of exercise should be considered as a possible explanation, as in our population only 30% of students performed enough physical activity to qualify for minimally active.

Our study presents the weaknesses inherent to transversal designs as far as the interpretation of causal factors is concerned. We did not include history of psychopathological disorders as part of our exclusion criteria, therefore, in theory we cannot rule out reverse causality; for instance it can be hypothesised that certain personality traits, associated with specific cognitive patterns, would favour heavier and earlier alcohol consumption. Large-scale longitudinal studies, such as the National Consortium on Alcohol and Neurodevelopment in Adolescence, recently started in the USA, are necessary to be able to confirm this association reliably and to evaluate the long-term consequences of alcohol consumption in adolescents and young adults. [52]

In our study, we defined BD following the National Institute on Alcohol Abuse and Alcoholism criteria (5 or more alcoholic drinks in the space of two hours (4 for females) in an average month), however other authors have proposed to adjust this cutoff based on differences in alcohol unit definitions among countries. In particular, Parada and colleagues proposed for Spain 6 drinks for males and 4 for females. [53] On this basis, our criteria might have been less demanding for men than for women, which might be an alternative explanation for the gender differences found in TMT A. A possible confounding factor in our analysis could be the consumption of other drugs. Cannabis use was more frequent in our BD population, and it has also been associated with cognitive alterations in young individuals. [54–56] In our study, 25.7% of students admitted consuming cannabis regularly, although this variable was not associated with TMT A or B (p-values 0.57 and 0.46 respectively). Additionally, our multivariate analysis was adjusted according to cannabis use. However, we cannot rule out the effect of other drugs not registered in the questionnaire or a certain under-estimation of cannabis consumption associated with alcohol consumption, due to the fact that some students might not declare that they consumed them. We consider it improbable that this could imply a significant bias because even though it is illegal, the use of cannabis is relatively accepted in our social context.

## Conclusions

Our results suggest that intermittent consumption of large quantities of alcohol in a short period of time is associated to a significant impact on the cognitive function in young adults, the most susceptible domain being executive functions and particularly cognitive flexibility. We were not able to find a statistically significant modulator effect of physical exercise nor APOE genotype. Women may be more susceptible to this damage and its effect could be accumulative at an early age. Although longitudinal studies are necessary in order to observe the evolution of these cognitive deficits and evaluate their consequences in the future, these results should be taken as a wake-up call in terms of the permissiveness that some societies show regarding adolescent alcohol consumption.

## Supporting Information

S1 Table Studies analysing the effects of binge drinking on young people using neuropsychological tests. (DOCX)

S2 Table Results of the neuropsychological tests in both APOE*4 carriers and non-carriers. (DOCX)

S3 Table Results of the neuropsychological tests according to gender. (DOCX)

S4 Table Results of the neuropsychological tests according to levels of physical activity. (DOCX)
