# Supplementary material for: Binge Drinking in Young University Students Is Associated with Alterations in Executive Functions Related to Their Starting Age
Source: PLoS One. 2016 Nov 18;11(11):e0166834. doi: 10.1371/journal.pone.0166834 (PMC5115818; doi:10.1371/journal.pone.0166834)
Supplement: S1 Table — (DOCX) [file pone.0166834.s001.docx]

| Author  **S1 Table: Studies analysing the effects of binge drinking on young people using neuropsychological tests.** | Sample Size | Age | Methods | Main results |
| --- | --- | --- | --- | --- |
| Crego (2009) | 95  BD (42)  Non-BD (53) | BD 18.9 ± 0.5  Non-BD 18.7 ± 0.5 | Alcohol Use Disorder Identification Test (AUDIT)  Event-related potentials (ERPs)  Visual “identical pairs” continuous performance task (IP-CPT) | Presence of electrophysiological differences between the BD and non-BD whilst carrying out a visual task with a heavy memory workload. Significant differences were not observed in terms of gender or other factors  . |
| Parada (2012) | 122  BD (62)  Non-BD (60) | BD 18.86 ± 0.55  Non-BD 18.7± 0.52 | Backward Digit Span and Backward Spatial Span ( WMS-III)  Self-Ordered Pointing Test (SOPT)  Letter Fluency  Zoo Map and Key Search (Behavioural Assessment of Dysexecutive Syndrome)  Wisconsin Card Sorting Test-3 (WCST-3). | BD are associated with worse results in tests evaluating executive functions.  The results do not support greater vulnerability in women to the effects of alcohol. |
| Hartley (2004) | 27  BD (14)  Non-BD(13) | BD 21.4 ± 0.6  Non-BD 20.85± 0.55 | Alcohol Use Questionnaire (AUQ)  Paced Auditory Serial Addition Test (PASAT)  Tests of episodic memory  Tests of executive (frontal lobe) function | Worse results in terms of attention, planning and episodic memory in the BD group.  No significant effects on the mental flexibility tests. |
| García-Moreno  (2008) | 62  High consumption (20)  Moderate consumption (20)  No consumption (22) | 18.82±1.10 | Encuesta Sobre Actitudes en la Juventud (ESAJ) (Spanish questionnaire regarding attitudes of young people)  Test de aprendizaje verbal España-Complutense (TAVEC) (Spain-Complutense verbal learning test)  Tower of Hanoi  Wechsler Memory Scale (digits)  Visio-spatial memory (Corsi block-tapping test)  Stroop colour and word test  Series recital (Numerical series): | Young people with high and moderate alcohol consumption performed worse in neuropsychological tasks such as Digits, Corsi or Stroop. |
| Heffernan (2009) | 50  BD (21)  Non-BD (29) | BD 18.7± 0.46  Non-BD 18.6 ± 0.48 | Recreational Drug Use  Questionnaire (modified version  of the University of East London)  Prospective and Retrospective Memory Questionnaire (PRMQ)  Prospective Remembering Video Procedure (PRVP)  Hospital Anxiety and Depression Scale (HADS) | Differences were not found between the group in the short and long term in the PRMQ.  The more alcohol is consumed, the worse the prospective memory is.  There is no correlation between the number of years spent drinking and the PRVP scores. |
| Scaife (2009) | 60  BD (30)  Non-BD (30) | BD 20.7 ± 2.97  Non-BD 22.3 ± 5.24 | Alcohol and drug use  Personality Trait measurements  Current mood measures  Spielberger State-Trait Anxiety Inventory  National Adult Reading Test (NART)  Paired Associates Learning task (PAL)  Spatial Working Memory task (SWM)  Intra/Extradimensional Shift task (IED)  Reaction Time Task (RTI) | The results showed differences in the female sex between BD and non-BD in the test evaluating mental attention and flexibility IED  Differences between groups in the test evaluating memory and visual learning PAL  The BD group obtained better results in the test evaluating reaction times |
| Squeglia (2011) | 95  BD (40)  Non-BD (55) | BD 17.95± 0.86  Non-BD 18.20 ± 0.96 | Substance use  Complex Figure copy/delay  Wechsler Abbreviated Scale of Intelligence (WASI) Block Design  WAIS-III Digits forward/backward  Digit Vigilance Test (DVT) (seconds)  WAIS-III Digit symbol  WRAT-3 Reading  Functional magnetic resonance imaging (fMRI) | Differences between genders in the BD group in frontal, temporal and cerebellar activation in the brain during the tasks evaluating spatial and work memory.  Women who binge drink turn out to be more vulnerable during adolescence to the effects of alcohol on a cognitive level. |
| Townshend  (2005) | 72  BD (38)  Non-BD (34) | BD 20.9 ± 2.6  Non-BD 20.9 ± 2.5 | Alcohol and Drug Use Questionnaires  Trait Measurements  Current Mood Measure*s*  National Adult Reading Test (NART)  Matching to Sample Visual Search task  Spatial Working Memory  The Vigilance Task for Adults from the Gordon Diagnostic System | Women who binge drink made more mistakes compared to the non-BD in the cognitive tests.  The BD were faster in the tests evaluating reaction times.  Mood also obtained worse results in the BD. |
| Sneider (2014) | 51  BD (22)  LD (light drinker) (29) | BD 22.1 ± 1.3  LD 21.5 ± 1.7 | Clinical measures  WASI Block Design subtest  Mental Rotation Task  Morris Water Maze Task (WMT)  California Verbal Learning Test (CVLT) | There are no significant differences between the groups, neither in terms of gender nor in the results of tests evaluating memory and visio-spatial and verbal learning.  Interaction was not observed between alcohol consumption and memory |
| Crego (2010) | 95  BD (42)  Non-BD (53) | BD 18.9 ± 0.5  Non-BD 18.7 ± 0.5 | AUDIT  Visual IP-CPT  Event-related potentials (ERPs)  Exact low-resolution brain electromagnetic tomography (eLORETA) | The BD showed hypoactivation of the right-hand anterior prefrontal cortex.  These findings may reveal alterations in the working memory of BD at an early age. |
| Day (2013) | 91 | 19.36 ± 0.77 | AUDIT  Breath alcohol concentration (BrAC)  Trail Making Test (TMT) A and B.  TMT composite | The results showed that the BrAC predicts performance in the TMT B but not with the TMT A.  There is a link between the years spent drinking and the results of both tests (TMT A and TMT B) |
