# Supplementary material for: Binge Drinking in Young University Students Is Associated with Alterations in Executive Functions Related to Their Starting Age
Source: PLoS One. 2016 Nov 18;11(11):e0166834. doi: 10.1371/journal.pone.0166834 (PMC5115818; doi:10.1371/journal.pone.0166834)
Supplement: S2 Table — (DOCX) [file pone.0166834.s002.docx]

|  | Non-*APOE**4 (N= 144) mean ±SD | *APOE**4 (N=23) mean ±SD | P-value |
| --- | --- | --- | --- |
| Logical memory WAIS-III (LMW) immediate recall | 23.88±6.14 | 23.48±6.15 | 0.77 |
| LMW delayed recall | 26.62±6.46 | 24.22±6.28 | 0.09 |
| CERAD immediate recall | 25.54±31.78 | 25.48±2.00 | 0.92 |
| CERAD deferred words | 9.03±0.93 | 9.04±1.43 | 0.96 |
| CERAD recognition list | 19.80±1.15 | 19.91±0.84 | 0.64 |
| Rey figure copy | 35.54±1.78 | 35.96±0.21 | 0.01 |
| Rey figure delayed visual memory | 24.32±5.70 | 25.00±6.28 | 0.60 |
| Direct digit span | 9.57±2.20 | 10.26±2.24 | 0.16 |
| Reverse digit span | 7.48±8.21 | 6.87±1.58 | 0.72 |
| Stroop test interference | 51.88±8.84 | 50.35±9.91 | 0.50 |
| Trail making test A | 18.59±6.04 | 17.43±4.39 | 0.38 |
| Trail making test B | 40.57±14.77 | 41.61±17.39 | 0.76 |

**S2 Table. Results of the neuropsychological tests in both APOE*4 carriers and non-carriers.**
