# Supplementary material for: Binge Drinking in Young University Students Is Associated with Alterations in Executive Functions Related to Their Starting Age
Source: PLoS One. 2016 Nov 18;11(11):e0166834. doi: 10.1371/journal.pone.0166834 (PMC5115818; doi:10.1371/journal.pone.0166834)
Supplement: S3 Table — (DOCX) [file pone.0166834.s003.docx]

|  | Women (N= 139) mean ±SD | Men (N=67) mean ±SD | P-value |
| --- | --- | --- | --- |
| Logical memory WAIS-III (LMW) immediate recall | 23.38±6.88 | 24.42±6.70 | 0.26 |
| LMW delayed recall | 26.56±6.39 | 25.68±6.68 | 0.37 |
| CERAD immediate recall | 25,63±2.51 | 25.36±3.53 | 0.52 |
| CERAD deferred words | 8.96±1.10 | 9.24±1.69 | 0.16 |
| CERAD recognition list | 19.92±0.32 | 19.67±1.65 | 0.23 |
| Rey figure copy | 35.61±1.32 | 35.60±1.88 | 0.84 |
| Rey figure delayed visual memory | 24.77±8.11 | 25.09±6.55 | 0.37 |
| Direct digit span | 9.73±2.22 | 9.76±2.13 | 0.92 |
| Reverse digit span | 6.83±2.20 | 8.52±11.84 | 0.25 |
| Stroop test interference | 52.04±8.44 | 51.90±9.22 | 0.91 |
| Trail making test A | 18.70±5.84 | 17.39±5.27 | 0.12 |
| Trail making test B | 41.12±14.97 | 39.88±13.21 | 0.57 |

**S3 Table. Results of the neuropsychological tests according to gender.**
