# Supplementary material for: Binge Drinking in Young University Students Is Associated with Alterations in Executive Functions Related to Their Starting Age
Source: PLoS One. 2016 Nov 18;11(11):e0166834. doi: 10.1371/journal.pone.0166834 (PMC5115818; doi:10.1371/journal.pone.0166834)
Supplement: S4 Table — (DOCX) [file pone.0166834.s004.docx]

|  | Insufficiently active (N= 145) mean ±SD | Minimally active (N=61) mean ±SD | P-value |
| --- | --- | --- | --- |
| Logical memory WAIS-III (LMW) immediate recall | 23.77±6.20 | 23.61±6.13 | 0.87 |
| LMW delayed recall | 26.28±6.61 | 26.26±6.23 | 0.98 |
| CERAD immediate recall | 25.50±3.00 | 25.64±2.60 | 0.76 |
| CERAD deferred words | 9.01±1.49 | 9.15±0.80 | 0.40 |
| CERAD recognition list | 19.85±0.82 | 19.82±1.30 | 0.83 |
| Rey figure copy | 35.57±1.67 | 35.75±1.11 | 0.44 |
| Rey figure delayed visual memory | 24.19±5.61 | 25.24±5.86 | 0.18 |
| Direct digit span | 9.58±2.21 | 10.11±2.10 | 0.11 |
| Reverse digit span | 7.43±8.20 | 7.25±2.42 | 0.86 |
| Stroop test interference | 51.61±8.60 | 53.00±8.86 | 0.33 |
| Trail making test A | 18.64±5.70 | 17.58±5.20 | 0.22 |
| Trail making test B | 41.70±14.72 | 38.62±13.05 | 0.16 |

**S4 Table. Results of the neuropsychological tests according to levels of physical activity.**
